# Quantitatively Evaluate the Improvement of Functional Cure for the Quality of Life of Chronic Hepatitis B Cases: Evidence from a Cross-Sectional Study in China

**DOI:** 10.3390/healthcare13202590

**Published:** 2025-10-14

**Authors:** Sihui Zhang, Zhiliang Gao, Hui Li, Yi Kang, Lei Fu, Xuebing Chen, Xiaoyuan Xu, Xinyue Chen, Hui Zhuang, Hui Zheng, Fuqiang Cui

**Affiliations:** 1 Department of Epidemiology and Biostatistics, School of Public Health, Peking University, Beijing 100191, China; 2 The Third Affiliated Hospital, Sun Yat-sen University, Guangzhou 510000, China; 3 The Second People’s Hospital of Yunnan Province, Kunming 650251, China; 4 Henan Provincial People’s Hospital, Zhengzhou 450000, China; 5 Xiangya Hospital, Central South University, Changsha 410008, China; 6 People’s Hospital of Deyang City, Deyang 618000, China; 7 Department of Infectious Diseases, Peking University First Hospital, Beijing 100034, China; 8 Department of Hepatology, Beijing Institute of Hepatology, Beijing You’an Hospital, Capital Medical University, Beijing 100052, China; 9 Department of Microbiology and Centre for Infectious Disease, School of Basic Medical Sciences, Peking University, Beijing 100191, China; 10 National Immunization Programme, Chinese Center for Disease Control and Prevention, Beijing 100050, China; 11 Department of Laboratorial Science and Technology & Vaccine Research Center, School of Public Health, Peking University, Beijing 100191, China; 12 Key Laboratory of Epidemiology of Major Diseases (Peking University), Ministry of Education, Beijing 100191, China; 13 Global Center for Infectious Disease and Policy Research & Global Health and Infectious Diseases Group, Peking University, Beijing 100191, China

**Keywords:** Hepatitis B, functional cure, health-related quality of life (HRQoL), quantitative analysis

## Abstract

**Background**: Functional cure of chronic hepatitis B (CHB) can be achieved with appropriate antiviral treatment. However, few studies have evaluated the added benefits of achieving functional cure. We aimed to conduct a quantitative analysis of the health-related quality of life (HRQoL) of CHB patients who achieved functional cure to provide evidence for economic analysis. **Methods**: We conducted a cross-sectional study in five provinces in China in 2021. The study population was recruited in hospitals and divided into three groups: functional cure, antiviral treatment, and healthy control group. Data were collected through face-to-face interviews using the Short Form-36 version 2. **Results**: 497 participants (163 with functional cure, 192 with antiviral treatment, and 142 with healthy control) were used in this study. The eight scale scores (physical function, role physical, bodily pain, general health, vitality, social functioning, role emotional, and mental health) and two summary scores (physical composite and mental composite) in the functional cure and healthy control groups were similar. Compared to the healthy control group, the general health scores in the functional cure group were worse with −0.052 (95% CI: −0.094, −0.010), and the antiviral treatment group had significantly worse scores with −0.127 (95% CI: −0.170, −0.083). The antiviral treatment group had lower vitality scores (β = −0.048, 95% CI: −0.089 to −0.007) and MCS scores (β = −0.023, 95% CI: −0.042, −0.003, *p* = 0.022) compared to the healthy control. The mental composite summary scores of all groups were >50 (*p* > 0.05). **Conclusions**: Health-related quality of life decreases with CHB disease progression. The results indicate that functional cure is associated with HRQoL levels comparable to those of the healthy population, both on the physical and psychological aspect, reinforcing the clinical value of this therapeutic goal.

## 1. Introduction

Hepatitis B virus (HBV) infection is a major public health problem globally. Despite the availability of antiviral therapy that could delay disease progression, it remains a significant cause of mortality, with deaths increasing from 820,000 in 2019 [1] to 1,100,000 in 2022 [2], while the global prevalence of chronic hepatitis B (CHB) infection declined from 296 million to 254 million during the same period, with approximately 80 million and 75 million of these residing in China [2,3,4]. Although the widespread implementation of universal hepatitis B vaccination, particularly in China, has successfully reduced the hepatitis B surface antigen (HBsAg) prevalence in the general population [2,5], the historical prevalence of HBV has left a large reservoir of chronic cases. Consequently, the morbidity and mortality burden remain substantial, shifting the clinical challenge towards the effective long-term management of existing infections to prevent disease progression. This underscores the critical need to evaluate treatment endpoints, such as functional cure, not only on serological or virological grounds but also on patient-centered outcomes like health-related quality of life.

Currently, long-term management, especially treatment, for CHB infections has greatly progressed. Antiviral therapies can effectively be used to treat CHB and reduce the risk of liver failure and liver cancer. However, antiviral therapy for hepatitis B patients needs to last for many years or even for life, which greatly affects the patient’s treatment compliance and their quality of life [6]. The optimal endpoint, which is a CHB-sterilizing cure or virologic cure, defined by the thorough clearance of both extrachromosomal covalently-closed-circular DNA (cccDNA) and chromosomally integrated viral DNA fragments, has rarely been achieved owing to the lack of curative strategies capable of eradicating cccDNA. However, achieving a functional cure or clinical cure, which is defined as sustained undetectable HBV DNA levels and HBsAg loss for half a year over a finite treatment period, is the ideal endpoint of treatment for CHB [7,8]. Despite the continued presence of cccDNA, its transcriptional activity is undetectable. Achieving functional cure has the potential to arrest the progression of liver disease to advanced stages [9], consequently lowering the incidence of liver-related complications and fatalities and enhancing long-term patient prognosis. This advancement would significantly reduce the financial strain on healthcare infrastructures and individual patients [10], while also supporting the global public health objective of eliminating chronic hepatitis B by diminishing the reservoir of individuals with chronic infection. In clinical practice, a combination of treatment medications consisting of nucleoside analogs and pegylated interferon (PEG IFN) has resulted in functional cure. Additionally, multiple targeted therapies are now being used in early human studies to achieve functional cure [11].

While the definition of functional cure is well-established, a significant evidence gap remains regarding its impact on patient-centered outcomes. Although antiviral treatment can improve the quality of life of CHB patients, it remains inferior to that of healthy individuals. Achieving functional cure has the potential to further enhance the quality of life of patients with CHB; however, due to the low proportion of hepatitis B patients who can achieve functional cure (0.6–10.5 per 100 person-years) [12], no research has been able to quantitatively evaluate the health-related quality of life (HRQoL) improvement of functional cure patients in the real world. A recent systematic review and meta-analysis [13] comprehensively summarized the impact of HBV infection on patients’ HRQoL globally; however, it did not include comparisons with functionally cured individuals. Other literature exhibits similar limitations. This gap impedes a comprehensive understanding of the clinical value of functional cure beyond sterilizing cure endpoints and limits its integration into health-economic evaluations and policy guidance.

Our study aimed to address this critical evidence gap by using a standard questionnaire to quantitatively evaluate HRQoL in patients who achieved functional cure compared to those in different CHB disease stages. These findings provide robust real-world evidence that informs clinicians, policy makers, and health economists. For clinicians, this evidence supports clearer communication with patients regarding the holistic benefits of treatment targets, thereby promoting shared decision-making, increasing the confidence in achieving a functional cure and improving the patients’ adherence to antiviral treatment. For policy makers, it offers a foundation for developing value-based treatment strategies that prioritize meaningful patient outcomes. Furthermore, these results supply essential parameters for refined cost-effectiveness analyses that incorporate HRQoL gains, ultimately supporting investment in curative therapies and advancing public health efforts toward HBV elimination.

## 2. Materials and Methods

### 2.1. Site Selection

This was a nation-wide cross-sectional study, conducted in the Guangdong, Henan, Hunan, Sichuan, and Yunnan provinces in China. In each province, we selected tertiary hospitals, which were recognized as the best hospitals for liver disease treatment locally, to recruit the target population. In other words, tertiary hospitals were exclusively selected to ensure adequate recruitment of the rare functionally cured persons and enable comprehensive HRQoL comparison across diverse CHB stages. While potentially limiting generalizability, for the primary objective of this study—to deeply characterize and contrast the HRQoL of patients with functional cure against other CHB groups—tertiary hospitals offered the optimal, and most logistically feasible, environment to ensure rigorous patient definition and data collection.

### 2.2. Study Population and Sample Size

Throughout the pre-defined recruitment period, all eligible patients who attended the outpatient clinics of the participating hepatology departments at each hospital were systematically invited to participate in the study. This approach minimized selection bias by the research team and ensured that the sample was representative of the patient population flowing through these specialized clinical centers during that time, rather than a convenience sample based on physician or researcher preference.

We recruited the subjects into three groups, and the inclusion and exclusion criteria of the target population in each group were as follows:

#### 2.2.1. Functional Cure

The inclusion criteria were as follows: ➀ those diagnosed functional cure for at least one year, according to The Expert Consensus on Clinical Cure (Functional Cure) of Chronic Hepatitis B, which was published by the Chinese Society of Infectious Disease, Chinese Society of Hepatology of Chinese Medical Association and its update version 2.0 [7,8]; ➁ ≥18 years old; and ➂ consent to participate in the investigation. The exclusion criteria were as follows: ➀ acute hepatitis, hepatocellular carcinoma (HCC), drug-induced liver disease, alcoholic liver disease, fatty liver disease, and autoimmune liver disease; ➁ hepatitis C or D, or CHB combined with hepatitis C or HIV infection; and ➂ other serious comorbidities.

#### 2.2.2. CHB with Antiviral Treatment Group

The inclusion criteria were as follows: ➀ those with CHB who were diagnosed in accordance with The Diagnostic Criteria for Viral Hepatitis B (WS299-2008); ➁ ≥18 years old; ➂ those who were being treated with oral antiviral drugs, including tenofovir (TDF) and entecavir (ETV), and ➃ consent to participate in the investigation. The exclusion criteria were same with the functional cure group.

#### 2.2.3. Healthy Control Group

The inclusion criteria were as follows: ➀ healthy population in the physical examination departments of the investigating hospitals, with age and sex matching with functional cure group; ➁ ≥18 years old; and ➂ consent to participate in the investigation. We further excluded those who had taken medication or those who had been ill one week before our study.

The sample size was calculated using the following empirical formula [14]:

(1) $$sample\;size\;=\;\left[Max\;\left(scales\right)\times \left(10-20\right)\right]$$

[Max (scales)] referred to the number of items in the dimension that contained the most questions among all dimensions. In this questionnaire, the value of [Max (scales)] was set to 9. “(10 − 20)” represented an empirical coefficient, meaning that each item required 10 to 20 respondents. Based on the theoretically calculated value, an additional 10% to 20% of the sample size was added to ensure that the final valid sample size still met the requirements.

For each group, the expected number of subjects was 99 (9 × 10 × 1.1) − 216 (9 × 20 × 1.2). Taking into account field work constraints, approximately 150 subjects would be enrolled for each disease status (or healthy control) group, requiring about 450 participants in total for all three groups.

### 2.3. Data Collection and Quality Control

The data were collected from April to August in 2021. The Chinese Short Form-36 version 2 (SF-36-V2) questionnaire was adopted to evaluate HRQoL, which has been extensively validated and widely used in Chinese populations, including patients with chronic hepatitis B and other liver diseases [15,16,17,18]. It contained 36 items that measured eight scales of health-related quality of life: physical function, role physical, bodily pain, general health, vitality, social functioning, role emotional, and mental health. The scores of the eight scales were aggregated into two summary scores: the physical composite summary and mental composite summary. The all scale scores were transformed based on the Chinese norms [19]. Face-to-face interviews were conducted by a team of specifically trained research nurses. To ensure standardization across all interviews, the research nurses underwent a structured training session that covered the objectives of the study, the precise meaning and purpose of each questionnaire item, and techniques for neutral probing to avoid influencing participants’ responses. All interviews were conducted in a private room within the clinic to ensure the confidentiality of the participants’ information. Furthermore, all collected data were anonymized immediately after collection, with questionnaires being identified only by a unique study ID rather than personal identifiers. Each subject completed the SF-36-V2 and provided sociodemographic and disease characteristic data. All questions in the questionnaire must be answered by the respondent themselves. Upon completion, another staff member will review the questionnaire to ensure there are no missing items or logical inconsistencies before concluding the survey. We double-entered the data into the EpiData (version 3.1, Denmark) software.

### 2.4. Statistical Analysis

For descriptive analysis, the categorical variables were described using frequency and percentage, while continuous variables are presented using the mean with standard deviation (SD). One-way ANOVA or Chi-squared test/Fisher exact tests were used to compare differences in socio-demographics among patient groups. The differences of SF-36-V2 scores were calculated and compared using the Kruskal–Wallis test for non-normally distributed continuous variables. If significant differences were found, multiple comparisons were conducted to further examine differences between groups using the Wilcoxon Rank-Sum Test. A Bonferroni correction for multiple comparisons was applied (α = 0.0167). To address potential confounding arising from the imbalanced distribution of baseline characteristics across groups, all demographic variables showing differences were included as covariates in the subsequent generalized linear models. A generalized liner model was used to investigate the effects of disease stage on HRQoL, β, and 95% confidence intervals (CI) using eight scales and two summary scores (physical composite summary and mental composite summary) as dependent variables by adjusting sociodemographic factors, including age group and region, and *p* value < 0.05 or 95%CI without 0 was considered statistically significant. All statistical analyses were performed using R 4.0.2 (R Core Team, Vienna, Austria).

### 2.5. Ethics Approval

The Peking University Biomedical Ethics Committee approved this study (IRB00001052-21011). Patient written informed consent was obtained at the time of enrolment. The privacy of our subjects was upheld.

## 3. Results

### 3.1. Subjects’ Characteristics

Table 1 showed the socio-demographic characteristics of patients overall and by groups. Among the 497 subjects enrolled in our study, 163 (32.8%), 192 (38.6%), and 142 (28.6%) were functional cure, CHB patients with antiviral treatment, and healthy control, respectively. Of them, 171 (34.4%), 147 (29.6%), and 179 (36.0%) were from the eastern region, middle region, and western region, respectively. The overall mean age of the total sample was 37.3 ± 8.7 years. The functional cure group had a mean age of 38.9 ± 8.6 years, the CHB patients undergoing antiviral treatment had a mean age of 38.7 ± 8.0 years, and the healthy control group had a mean age of 33.5 ± 8.8 years. The majority of participants were male across all groups (283, 57.1%), and over half had a high school education or below (288, 57.9%). Most participants were married (414, 83.3%). Significant differences (*p* < 0.05) were identified for region and age group using the Chi-squared test. No significant differences were found in gender, education, or marital status, with marital status analyzed using Fisher’s exact test.

### 3.2. Adjusted and Unadjusted Results on Eight Health Domains Scores

**Figure 1:**
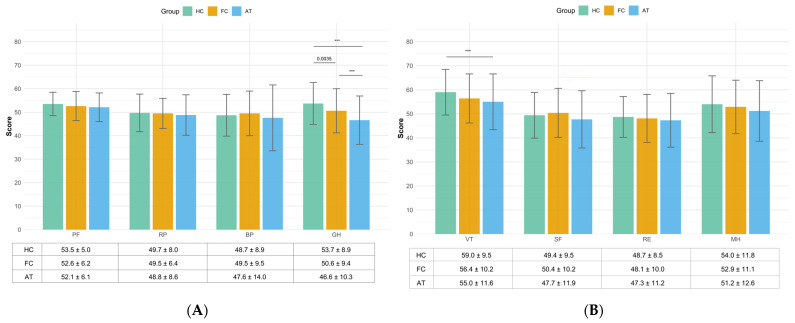
Unadjusted results on eight health domains scores among the HC, FC and AT groups in China in 2021. (**A**) The results of physical functioning (PF), role physical (RP), bodily pain (BP), general health (GH); (**B**) the results of vitality (VT), social functioning (SF), role-emotional (RE), and mental health (MH). ***: *p* < 0.001. Note: HC, healthy control group; FC, functional cure group; AT: CHB patients with antiviral treatment group.

There were no significant differences among the eight scale scores except for general health domains. Figure 1A,B compared the scores across eight domains including physical functioning, role physical, bodily pain, general health, vitality, social functioning, role emotional, and mental health among three groups: healthy controls, functional cure, and antiviral treatment. In the first set of domains, significant differences were observed in general health domain (*p* < 0.001), with the healthy control group scoring significantly higher than both the functional cure and antiviral treatment groups (*p* < 0.001), functional cure group scoring significantly higher than antiviral treatment group (*p* = 0.0035). Similarly, in the second set of domains, the vitality domain showed a significant difference (*p* < 0.001), with the healthy control group (59.0 ± 9.5) outperforming the antiviral treatment (55.0 ± 11.6) group, but no significant differences compared to the functional cure group.

According to the generalized linear model analysis adjusting the age group and region (Table 2), in the first set of domains, a significant association was identified for the general health domain. Compared to the healthy control group, both the functional cure group (β = −0.052, 95% CI: −0.094, −0.010, *p* = 0.015) and the antiviral treatment group (β = −0.127, 95% CI: −0.170, −0.083, *p* < 0.001) showed statistically significant reductions in general health scores. In summary, after adjusting for region and age, the patient group was the only factor significantly associated with general health, with both the functional cure and antiviral treatment groups reporting lower scores compared to healthy controls. However, the reduction in the functional cure group was not as pronounced as that in the antiviral treatment group. No other domains exhibited statistically significant relationships with any of the independent variables studied.

In the second set of domains, for the vitality domain, a statistically significant association was found for the antiviral treatment group (β = −0.048, 95% CI: −0.089 to −0.007, *p* = 0.023), indicating significantly lower vitality scores compared to the healthy control group. In summary, among these four mental health-related domains, only vitality showed a statistically significant difference, with the antiviral treatment group reporting significantly lower scores compared to healthy controls. The other three domains—social functioning, role emotional, and mental health—showed no significant associations with any of the independent variables after adjustment.

### 3.3. The Results of Two Summary Measures—Physical Component Summary and Mental Component Summary Scores

**Figure 2:**
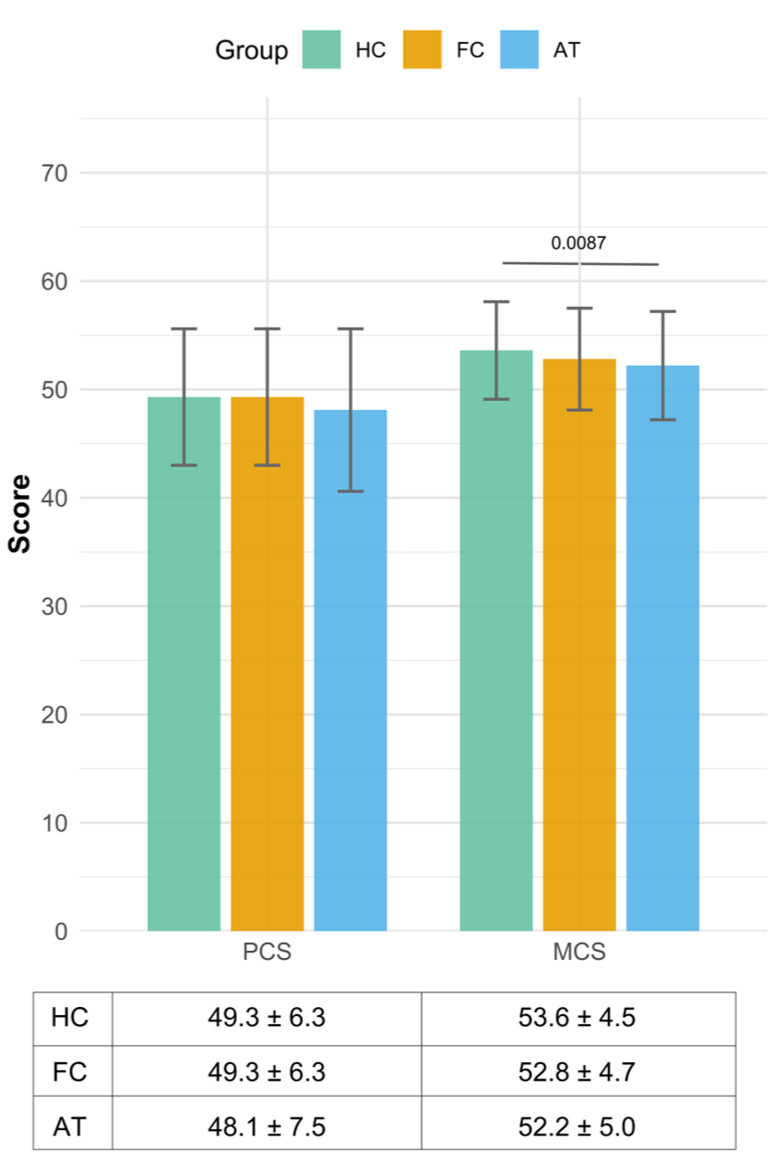
Unadjusted results of two summary measures—physical composite summary and mental composite summary scores in China in 2021. Note: HC, healthy control group; FC, functional cure group; AT: CHB patients with antiviral treatment group; PCS, physical composite summary; MCS, mental composite summary.

Based on Figure 2, the physical component summary and mental component summary scores were compared among the healthy control, functional cure, and antiviral treatment groups. No statistically significant difference was observed between the healthy control and functional cure groups in either physical composite summary or mental composite summary scores, with identical physical composite summary values (49.3 ± 6.3) and only a minimal difference of 0.8 in mental composite summary (healthy control: 53.6 ± 4.5 vs. functional cure: 52.8 ± 4.7), indicating that the quality of life in the functional cure group reached a level comparable to healthy controls in both the physical and mental dimensions. In contrast, significant differences were evident when comparing the antiviral treatment group with the healthy control group on mental composite summary scores. A more pronounced difference was observed in the mental composite summary score, where the antiviral treatment group’s score (52.2 ± 5.0) was 1.4 points lower than the healthy control group (*p* = 0.0087).

Based on the results of the generalized linear model by adjusting age group and region, the associations between independent variables and the two dependent variables, physical component summary and mental component summary, were presented in Table 3.

For physical component summary scores, none of the independent variables showed a statistically significant association. Compared to the Eastern region, neither the Middle (β = 0.001, 95% CI: −0.031, 0.035, *p* = 0.948) nor the Western region (β = 0.013, 95% CI: −0.017 to 0.042, *p* = 0.395) demonstrated a significant effect. Similarly, age group (β = −0.017, 95% CI: −0.042, 0.008, *p* = 0.201) was not a significant predictor. In terms of group comparison, using healthy control as the reference, neither functional cure (β = 0.012, *p* = 0.436) nor antiviral treatment (β = −0.009, *p* = 0.559) reached statistical significance.

For mental composite summary, most variables also showed no significant impact. However, the group variable revealed a notable finding: compared to healthy control, the antiviral treatment group was significantly associated with lower mental composite summary scores (β = −0.023, 95% CI: −0.042, −0.003, *p* = 0.022). No other factors, including region, age, or functional cure group status, were significantly associated with mental composite summary outcomes.

## 4. Discussion

To the best of our knowledge, this was the first study that evaluated the quality of life of CHB patients who achieved functional cure. The findings suggested that functional cure for CHB patients could improve the quality of life to the level of healthy people, both physically and psychologically. This highlighted a distinct pattern in HRQoL among the three groups. Although general health and vitality emerged as key areas where impairments persist even after clinical intervention, for patients with functional cure, the degree is relatively low.

Firstly, the significant reduction in general health scores in both the functional cure and antiviral treatment groups compared to the healthy control group suggests that perceived overall health remains compromised despite successful functional cure or ongoing treatment. Notably, the smaller coefficient in the functional cure compared to the antiviral treatment group indicates that functional cure attenuates but does not fully normalize this deficit. Currently, identifying an absolute cure is not an achievable goal for CHB treatment because of its complexities [20]. In 2015, China first described the concept of functional cure in the Chronic Hepatitis B Prevention and Treatment Guidelines [21]. The American Academy of Liver Diseases (AASLD), and the European Society of Liver Diseases (EASL) reached a consensus on this view in 2016 [22]. Functional cure has become a feasible goal of hepatitis B treatment, which has caused the most concern regarding clinical practices at present [23,24]. Previous studies suggested that the HRQoL of liver disease patients was significantly lower than that of healthy people, in recent years, the research on HRQoL of chronic liver disease patients caused by different etiology or in different disease status, and how to improve the quality of life has become a hot topic [16,25,26,27]. Apart from the impact on physical function, the psychological burden of liver disease patients is also heavy, especially for CHB patients, and this can lead to social problems [28,29]. In addition, the majority of CHB patients require antiviral therapy for years, which greatly affects their compliance with treatment. A recent qualitative study reported that patients with CHBs were eager to achieve functional cure, allowing therapy to be removed while maintaining effective suppression of infection and reversal of liver damage [30].

Secondly, in the mental health cluster, vitality was significantly lower only in the antiviral treatment group, implying that fatigue or reduced energy levels are particularly associated with ongoing treatment burdens. The lack of significant differences in other domains is encouraging, indicating that functional recovery is sufficient to restore much of the patients’ psychological and social well-being. Patients with CHB have a variety of disease processes. Numerous studies have explored the relationship between CHB clinical stage and HRQoL. Several studies reported that quality of life decreased as the disease progressed; however, according to a recent study, after 5 years of ETV treatment, HRQoL significantly improved in patients [16,25,31,32].

Thirdly, the most notable result is that individuals in the functional cure group demonstrated physical composite summary and mental composite summary scores that were statistically indistinguishable from those of healthy controls. This strongly suggests that achieving functional cure can restore overall physical and mental well-being to a level comparable with the general healthy population, representing a highly encouraging outcome for clinical management and patient prognosis. In contrast, the antiviral treatment group showed a statistically significant reduction in mental health summary scores, which indicated that eligible patients should receive antiviral therapy as early as possible. A study from Thailand indicated that later stages of CHB compromised HRQoL [33]. In black African patients with CHB, the study also investigated HRQoL impairment [34]. However, research from India suggested that antiviral therapy can improve a wide range of physical and mental health associated with CHB [35]. Our results confirmed that the physical and psychological functions of the functional cure group were greatly improved, and almost reached the level of the healthy population. This could help further improve the confidence and compliance of patients who are currently being treated for CHB. This emphasized the importance of achieving functional cure for CHB, suggesting that this might be a way to reduce the disease burden and solve the social problems caused by HBV infection. In addition, achieving functional cure can be a key parameter for future economic evaluation of new strategies or medications that aim to achieve functional cure [36].

These results highlight the holistic benefit of achieving functional cure, which appears to normalize both the physical and mental health components of quality of life. For those undergoing antiviral treatment, the findings suggest a need for integrated care models that incorporate mental health support to address the significant gap in psychological well-being, such as counseling, stress management, and mental health screening. Future research should explore the specific determinants of mental health in these patients to develop targeted interventions aimed at improving overall quality of life during treatment.

This national representative study was supported by multiple-center design, a large sample size and empirical data. It has several limitations. First, the nationwide cross-sectional design was chosen as the most efficient and feasible method to achieve the study’s primary objective. However, we acknowledged the inherent limitation of this design: it captured data from a single time point and therefore cannot establish temporal sequence or causality. While we can report associations, causal inferences cannot be made from these findings alone. Second, the assessment of HRQoL using the eight scales was conducted only at a single time point. The absence of longitudinal data prevents any analysis of changes in these outcomes over time, particularly for the clinical subgroups such as the antiviral treatment and functional cure groups. Future studies incorporating serial measurements would provide valuable insights into the trajectory of quality of life in these populations. Third, the study was unable to continuously monitor ALT levels, HBV-DNA load, and HBsAg quantification in patients with CHB, and therefore could not assess the impact of these dynamic indicators on patients’ HRQoL. Also, it was not possible to determine the liver disease staging of the study participants. Fourth, all of the subjects in our study were outpatients, which might have resulted in selection bias for those in a stable disease stage with mild symptoms. This might have resulted in an overestimation of the HRQoL scores. Finally, the exclusive reliance on tertiary hospitals may introduce selection bias into the study. Individuals seeking care at these institutions often demonstrate greater health awareness and tend to originate from higher socioeconomic and educational backgrounds, which may inflate self-reported ratings of mental and physical health relative to populations in non-tertiary settings. To improve the generalizability and validity of future research, it would be advantageous to incorporate a more diverse array of healthcare institutions across different levels of the hospital system.

## 5. Conclusions

We found that HRQoL decreased as CHB progressed. Our study indicated that achieving functional cure for CHB cases could improve their quality of life to a level similar to that of healthy people, both in physical and psychological aspects. The findings reaffirmed the importance of treatment for CHB patients, illustrating that scaling up the treatment and achieving functional cure can be added value to survival and quality of life among those CHBs. To further validate and extend these results, future longitudinal studies are warranted to track dynamic changes in clinical and patient-reported outcomes over time. Additionally, interventional trials examining integrated care models—such as combining antiviral therapy with psychosocial support—could help improve holistic health outcomes in chronic hepatitis B patients, especially those yet to achieve functional cure.

## Figures and Tables

**Table 1:** Demographic characteristics of the study populations in the five provinces of China, 2021.

Characteristics	Total (N = 497)	Functional Cure (n = 163)	CHB Patients with Antiviral Treatment (n = 192)	Healthy Control (n = 142)
Region *				
East	171 (34.4)	50 (30.7)	75 (39.1)	46 (32.4)
Middle	147 (29.6)	51 (31.3)	46 (24.0)	50 (35.2)
West	179 (36.0)	62 (38.0)	71 (37.0)	46 (32.4)
**Gender**				
Male	283 (57.1)	100 (61.3)	128 (66.7)	86(61.0)
Female	213 (42.9)	63 (38.7)	64 (33.3)	55 (39.0)
Age group * (Mean ± SD)	37.3 ± 8.7	38.9 ± 8.6	38.7 ± 8.0	33.5 ± 8.8
<40 years	285 (57.3)	80 (49.1)	100 (52.1)	105 (73.9)
≥40 years	212 (42.7)	83 (50.9)	92 (47.9)	37 (26.1)
**Education**				
High school and below	288 (57.9)	93 (57.1)	111 (57.8)	84 (59.2)
Graduate	209 (42.1)	70 (42.9)	81 (42.2)	58 (40.8)
**Marriage**				
Unmarried	74 (14.9)	20 (12.3)	28 (14.6)	26 (18.3)
Married	414 (83.3)	140 (85.9)	162 (84.4)	112 (78.9)
Divorced/widowed	9 (1.8)	3 (1.8)	2 (1.0)	4 (2.8)

* Significant difference among three patient groups (*p*  <  0.05); Region, Gender, Age group and Education: using Chi-squared test; Marriage: using Fisher exact test; Gender information missing for one case.

**Table 2:** Generalized linear model adjusted age group and region analysis on eight health domains scores in China in 2021.

Dependent Variable	Independent Variable Entered in the Model	Standardized Coefficient	95% CI	*p* Value
**Physical functioning**	**Region**			
	East	Reference		
	Middle	0.012	−0.016, 0.040	0.405
	West	0.018	−0.007, 0.044	0.158
	**Age group**			
	<40 years	Reference		
	≥40 years	−0.015	−0.038, 0.007	0.176
	**Group**			
	Healthy control	Reference		
	Functional cure	−0.010	−0.043, 0.014	0.316
	CHB with antiviral treatment	−0.005	−0.036, 0.016	0.459
**Role physical**	**Region**			
	East	Reference		
	Middle	−0.016	−0.051, 0.02	0.388
	West	−0.005	−0.038, 0.028	0.756
	**Age group**			
	<40 years	Reference		
	≥40 years	0.002	−0.026, 0.03	0.898
	**Group**			
	Healthy control	Reference		
	Functional cure	−0.003	−0.036, 0.031	0.865
	CHB with antiviral treatment	−0.007	−0.024, 0.046	0.546
**Bodily pain**	**Region**			
	East	Reference		
	Middle	−0.037	−0.083, 0.009	0.113
	West	0	−0.045, 0.045	0.995
	**Age group**			
	<40 years	Reference		
	≥40 years	−0.006	−0.046, 0.034	0.768
	**Group**			
	Healthy control	Reference		
	Functional cure	0.028	−0.019, 0.075	0.244
	CHB with antiviral treatment	−0.001	−0.049, 0.05	0.984
**General health**	**Region**			
	East	Reference		
	Middle	−0.003	−0.047, 0.040	0.878
	West	0.029	−0.012, 0.071	0.166
	**Age group**			
	<40 years	Reference		
	≥40 years	−0.015	−0.051, 0.020	0.399
	**Group**			
	Healthy control	Reference		
	Functional cure	−0.052	−0.094, −0.010	0.015
	CHB with antiviral treatment	−0.127	−0.170, −0.083	<0.001
**Vitality**	**Region**			
	East	Reference		
	Middle	−0.023	−0.062, 0.015	0.232
	West	−0.005	−0.047, 0.036	0.797
	**Age group**			
	<40 years	Reference		
	≥40 years	0.008	−0.026, 0.042	0.642
	**Group**			
	Healthy control	Reference		
	Functional cure	−0.030	−0.070, 0.011	0.151
	CHB with antiviral treatment	−0.048	−0.089, −0.007	0.023
**Social functioning**	**Region**			
	East	Reference		
	Middle	−0.016	−0.064, 0.033	0.525
	West	−0.001	−0.047, 0.044	0.958
	**Age group**			
	<40 years	Reference		
	≥40 years	−0.011	−0.05, 0.028	0.578
	**Group**			
	Healthy control	Reference		
	Functional cure	0.015	−0.009, 0.024	0.183
	CHB with antiviral treatment	−0.034	−0.045, 0.052	0.174
**Role emotional**	**Region**			
	East	Reference		
	Middle	−0.035	−0.082, 0.013	0.152
	West	−0.018	−0.064, 0.027	0.433
	**Age group**			
	<40 years	Reference		
	≥40 years	0.012	−0.027, 0.050	0.560
	**Group**			
	Healthy control	Reference		
	Functional cure	−0.001	−0.043, 0.053	0.837
	CHB with antiviral treatment	−0.003	−0.048, 0.047	0.973
**Mental health**	**Region**			
	East	Reference		
	Middle	−0.046	−0.096, 0.003	0.067
	West	−0.002	−0.05, 0.047	0.949
	**Age group**			
	<40 years	Reference		
	≥40 years	0.011	−0.031, 0.053	0.617
	**Group**			
	Healthy control	Reference		
	Functional cure	−0.006	−0.057, 0.045	0.816
	CHB with antiviral treatment	−0.029	−0.081, 0.024	0.287

**Table 3:** Generalized linear model adjusted age group and region analysis on two summary measures—physical composite summary and mental composite summary scores in China in 2021.

Dependent Variable	Independent Variable Entered in the Model	Standardized Coefficient	95% CI	*p* Value
**Physical composite summary**	**Region**			
	East	Reference		
	Middle	0.001	−0.031, 0.033	0.948
	West	0.013	−0.017, 0.042	0.395
	**Age group**			
	<40 years	Reference		
	≥40 years	−0.017	−0.042, 0.009	0.201
	**Group**			
	Healthy control	Reference		
	Functional cure	0.012	−0.019, 0.044	0.436
	CHB with antiviral treatment	−0.009	−0.041, 0.022	0.559
**Mental composite summary**	**Region**			
	East	Reference		
	Middle	−0.013	−0.032, 0.005	0.156
	West	−0.003	−0.023, 0.017	0.782
	**Age group**			
	<40 years	Reference		
	≥40 years	0.0003	−0.016, 0.017	0.975
	**Group**			
	Healthy control	Reference		
	Functional cure	−0.001	−0.019, 0.022	0.899
	CHB with antiviral treatment	−0.023	−0.042, −0.003	0.022

## Data Availability

The dataset is available on reasonable request from the corresponding author due to ethical restrictions.

## Abbreviations

The following abbreviations are used in this manuscript:

CHB	Chronic hepatitis B
HRQoL	Health-related quality of life
HCC	Hepatocellular carcinoma
HBV	Hepatitis B virus
SF-36-V2	Chinese Short Form-36 version 2
